# Supplementary material for: Tissue resident and follicular Treg cell differentiation is regulated by CRAC channels
Source: Nat Commun. 2019 Mar 12;10:1183. doi: 10.1038/s41467-019-08959-8 (PMC6414608; doi:10.1038/s41467-019-08959-8)
Supplement: Supplementary file 1 — Supplementary Information [file 41467_2019_8959_MOESM1_ESM.pdf]

# Supplementary Information

## Tissue resident and follicular Treg cell differentiation is regulated by CRAC channels

Vaeth et al.

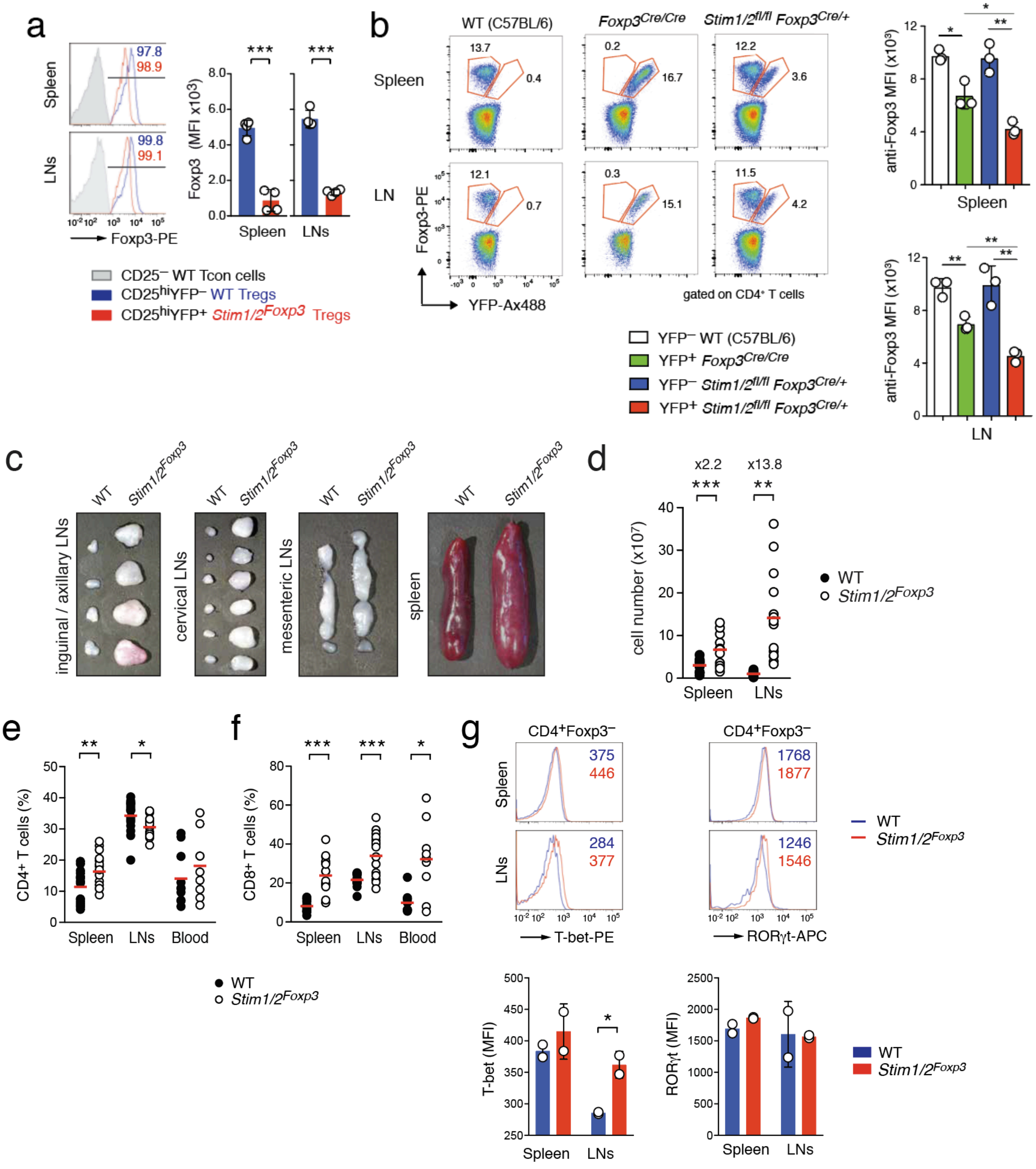


**Supplementary Figure 1. Treg-specific deletion of STIM1 and STIM2 causes splenomegaly and lymphadenopathy. (a)** Analysis of Foxp3 protein expression in Treg cells of male WT and *Stim1/2*$^{Foxp3}$ mice; mean ± SEM of 4 mice. **(b)** Analysis of Foxp3 protein expression in CD4$^{+}$ Treg cells from the spleen and LNs of female C57BL/6 wildtype (WT), *Foxp3*$^{YFPCre/YFPCre}$ and *Stim1/2*$^{fl/fl}$ *Foxp3*$^{YFPCre/+}$ mice. Representative flow cytometry plots and means ± SD from 3 mice per group. **(c)** Representative pictures of spleen as well as inguinal, axillary, cervical and mesenteric LNs of male WT and *Stim1/2*$^{Foxp3}$ mice. **(d)** Cellularity of spleen and all LNs of male WT and *Stim1/2*$^{Foxp3}$ mice; mean of 13-14 mice. **(e,f)** Frequency of CD4$^{+}$ **(e)** and CD8$^{+}$ **(f)** T cells in spleen, LNs and blood of male WT and *Stim1/2*$^{Foxp3}$ mice; mean of 9-16 mice. Each dot in b, c and d represents one mouse. **(g)** Analysis of T-bet and RORγt protein levels in CD4$^{+}$ T cells. Representative flow cytometry plots and means ± SEM of 2-3 mice. Statistical analysis in a,b,d-g by unpaired Student's t-test. *, p<0.05; **, p<0.01, ***, p<0.001.

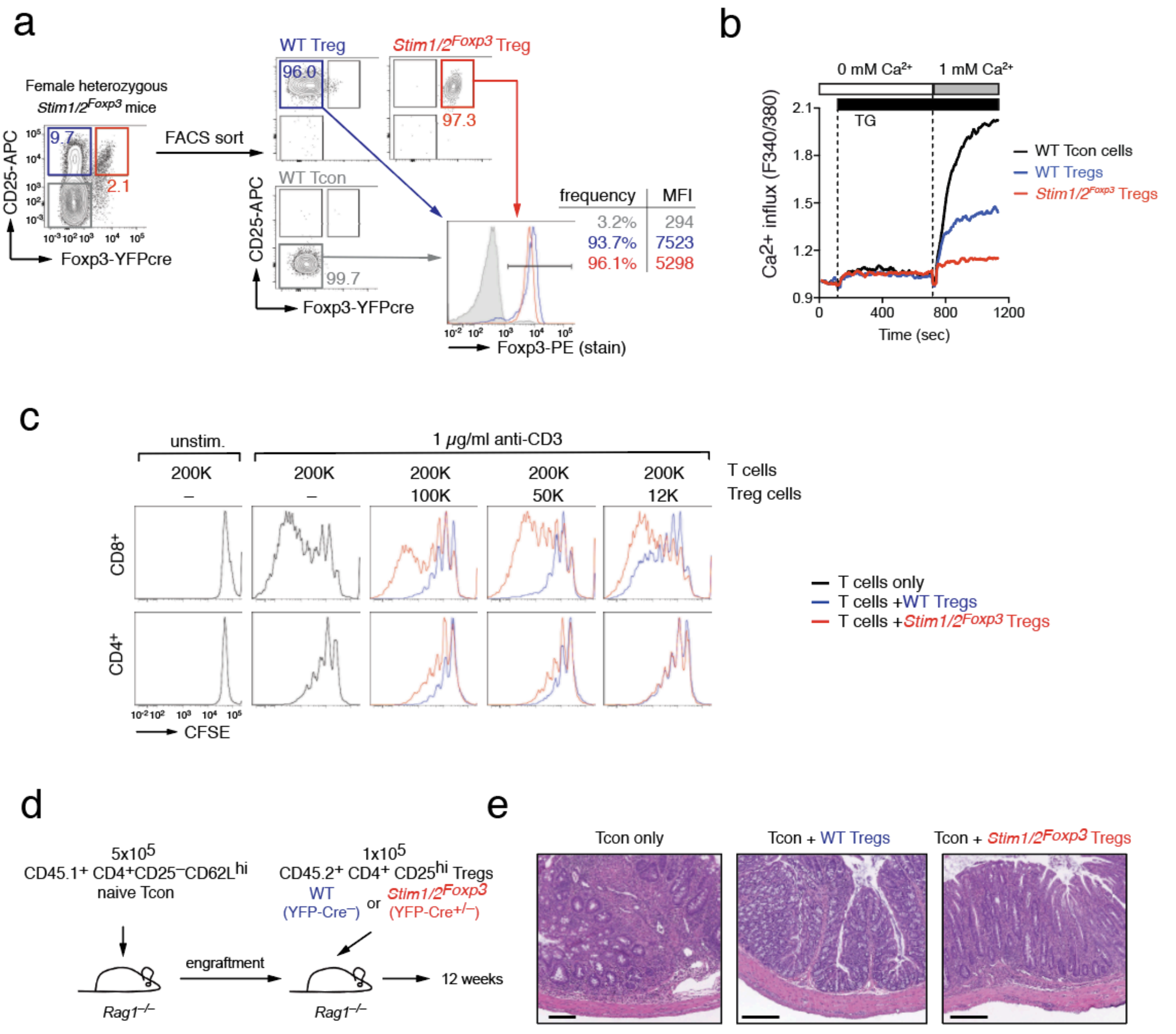


**Supplementary Figure 2. STIM1 and STIM2 control the immune-suppressive function of Treg cells. (a)** Gating strategy to isolate WT and *Stim1/2*-deficient Foxp3$^+$ Treg cells from female heterozygous *Stim1/2*$^{Foxp3}$ mice. **(b)** Analysis of SOCE in CD4$^+$CD25$^-$YFP$^-$ conventional T cells (Tcon), CD4$^+$CD25$^+$YFP$^-$ WT Tregs and CD4$^+$CD25$^+$YFP$^+$ Treg cells isolated by flow cytometric cell sorting from the spleen and LNs of female *Stim1/2*$^{fl/fl}$ *Foxp3*$^{YFPCre/+}$ mice. 1x10$^5$ cell were loaded with Fura-2 and stimulated with thapsigargin (TG) in Ca$^{2+}$ free Ringer solution followed by addition of 1 mM Ca$^{2+}$. Data are representative of 3 mice. **(c)** Suppression of CD4$^+$ and CD8$^+$ T cell proliferation by WT and *Stim1/2*-deficient Treg cells *in vitro*. 2x10$^5$ CFSE-labeled T cells were co-cultured with 1.2x10$^4$, 5x10$^4$ or 1x10$^5$ WT or *Stim1/2*-deficient Treg cells isolated from female heterozygous *Stim1/2*$^{Foxp3}$ mice and stimulated with anti-CD3. Proliferation of CD4$^+$ and CD8$^+$ T cells was analyzed 3 days later by flow cytometry. Data representative of 2 experiments. **(d)** Suppression of CD4$^+$ T cells by WT and *Stim1/2*-deficient Treg cells *in vivo*. Adoptive transfer of 5x10$^5$ naïve CD45.1$^+$CD4$^+$ Tcon cells to induce inflammatory bowel disease (IBD) in lymphocyte-deficient *Rag1*$^{-/-}$ host mice followed by transfer of 1x10$^5$ WT or *Stim1/2*-deficient Treg cells into the same host mice 2-4 weeks later. **(e)** Representative H&E stained colon sections of *Rag1*$^{-/-}$ host mice 12 weeks after transfer of Tcon cells only or Tcon cells together with WT or *Stim1/2*-deficient Treg cells. Images are representative of 3 mice analyzed per cohort.

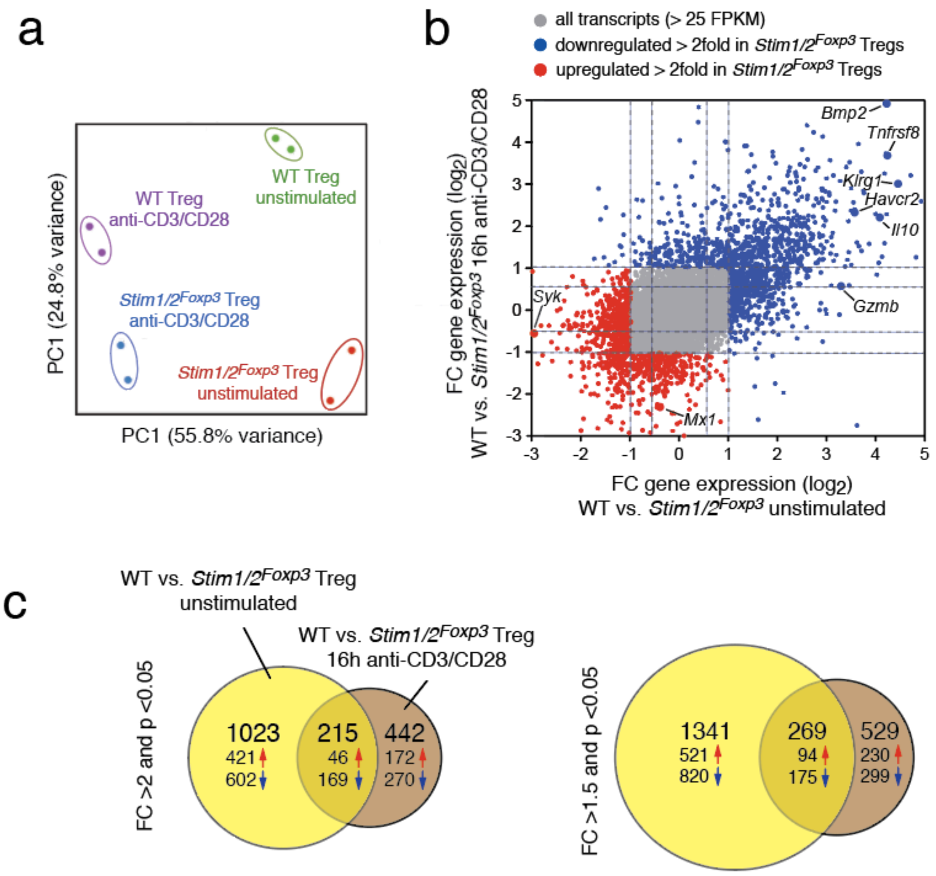


**Supplementary Figure 3. Transcriptome analysis of WT and *Stim1/2*-deficient Treg cells.** Transcriptional profiling of WT and *Stim1/2*-deficient Treg cells using RNA-sequencing. Spleen and LNs of 12-16 female hemizygous *Stim1/2$^{Foxp3}$* mice were pooled to isolate YFP$^{-}$ and YFP$^{+}$ Treg cells. WT and *Stim1/2*-deficient Treg cells used for RNA-sequencing were > 93% positive for Foxp3 after sorting (compare with gating strategy shown in Supplementary Figure 2a). **(a)** Principle component analysis (PCA) of unstimulated and anti-CD3/CD28 stimulated WT and *Stim1/2*-deficient Treg cells based on RNA-sequencing data. **(b)** Fold change of differentially expressed genes (DEG) of unstimulated and anti-CD3/CD28 stimulated WT and *Stim1/2*-deficient Treg cells; down- and upregulated genes are depicted in blue and red, respectively. **(c)** Venn diagrams of DEG that are expressed > 2 fold (left panel) and > 1.5 fold (right panel) in unstimulated and anti-CD3/CD28 stimulated WT versus *Stim1/2*-deficient Treg cells. Numbers of genes down- and upregulated are indicated by blue and red arrows, respectively.

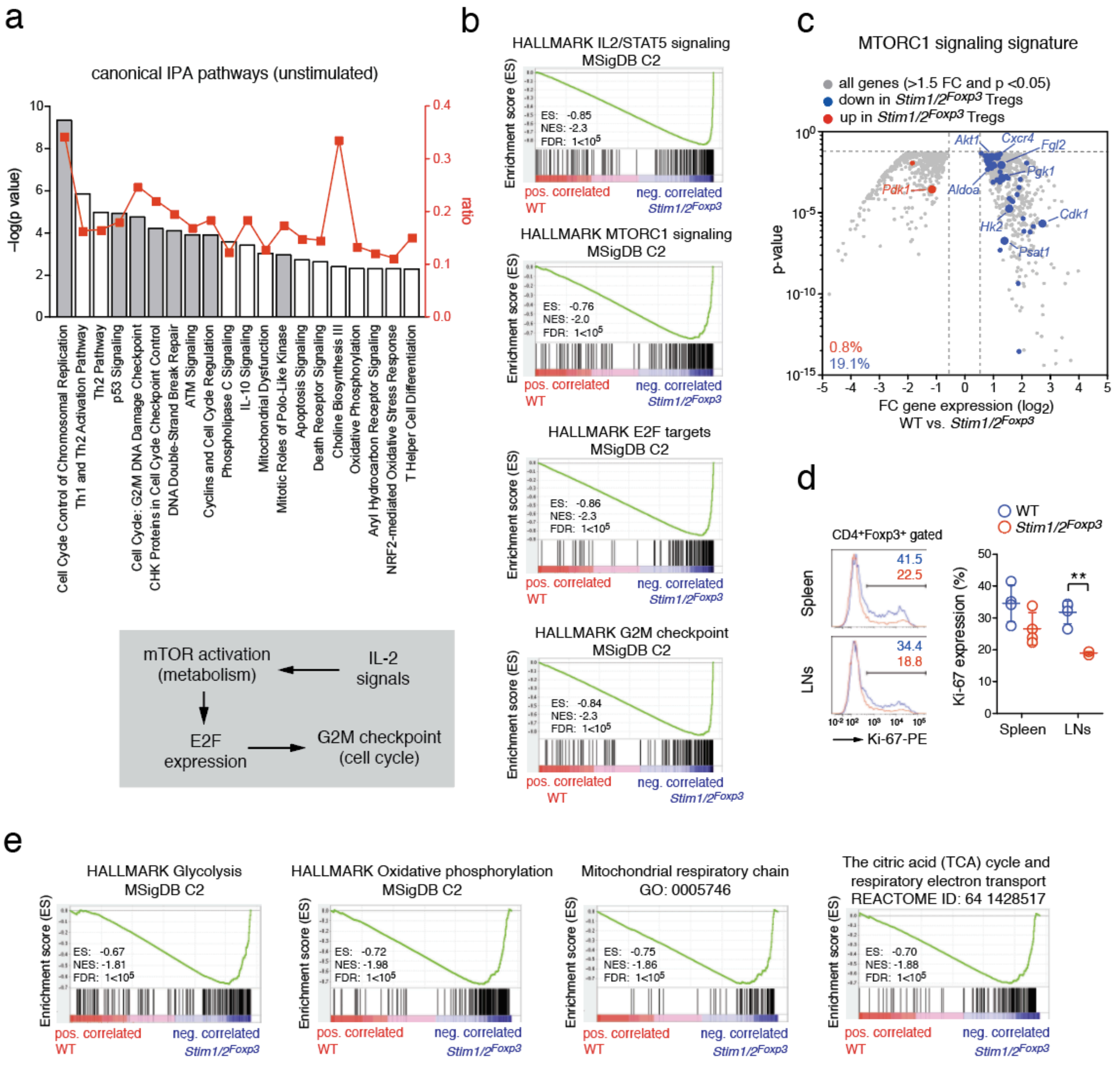


**Supplementary Figure 4. SOCE controls IL-2 and mTOR signaling in Treg cells. (a)** Canonical pathway analysis (IPA) of differentially expressed genes (DEG) in unstimulated WT *versus Stim1/2*-deficient Treg cells. **(b)** Top four enriched molecular signatures by gene set enrichment analysis (GSEA) of DEG in WT *versus Stim1/2*-deficient Treg cells. **(c)** Volcano plot of DEG (grey) in unstimulated WT and *Stim1/2*-deficient Treg cells overlaid with up- (red) or downregulated (blue) genes of the 'MTORC1 signaling signature' (Liberzon et al., 2015). **(d)** Analysis of cell cycle (Ki-67) in Foxp3$^{+}$ Treg cells isolated from the spleen and LNs of male WT and *Stim1/2*$^{Foxp3}$ mice by flow cytometry; mean ± SEM of 4 mice. **(e)** Identification of metabolic signatures by GSEA using DEG in unstimulated WT (YFP$^{-}$) *versus Stim1/2*-deficient (YFP$^{+}$) Treg cells from female hemizygous *Stim1/2*$^{Foxp3}$ mice. Statistical analysis in d by unpaired Student's t-test. **, p<0.01.

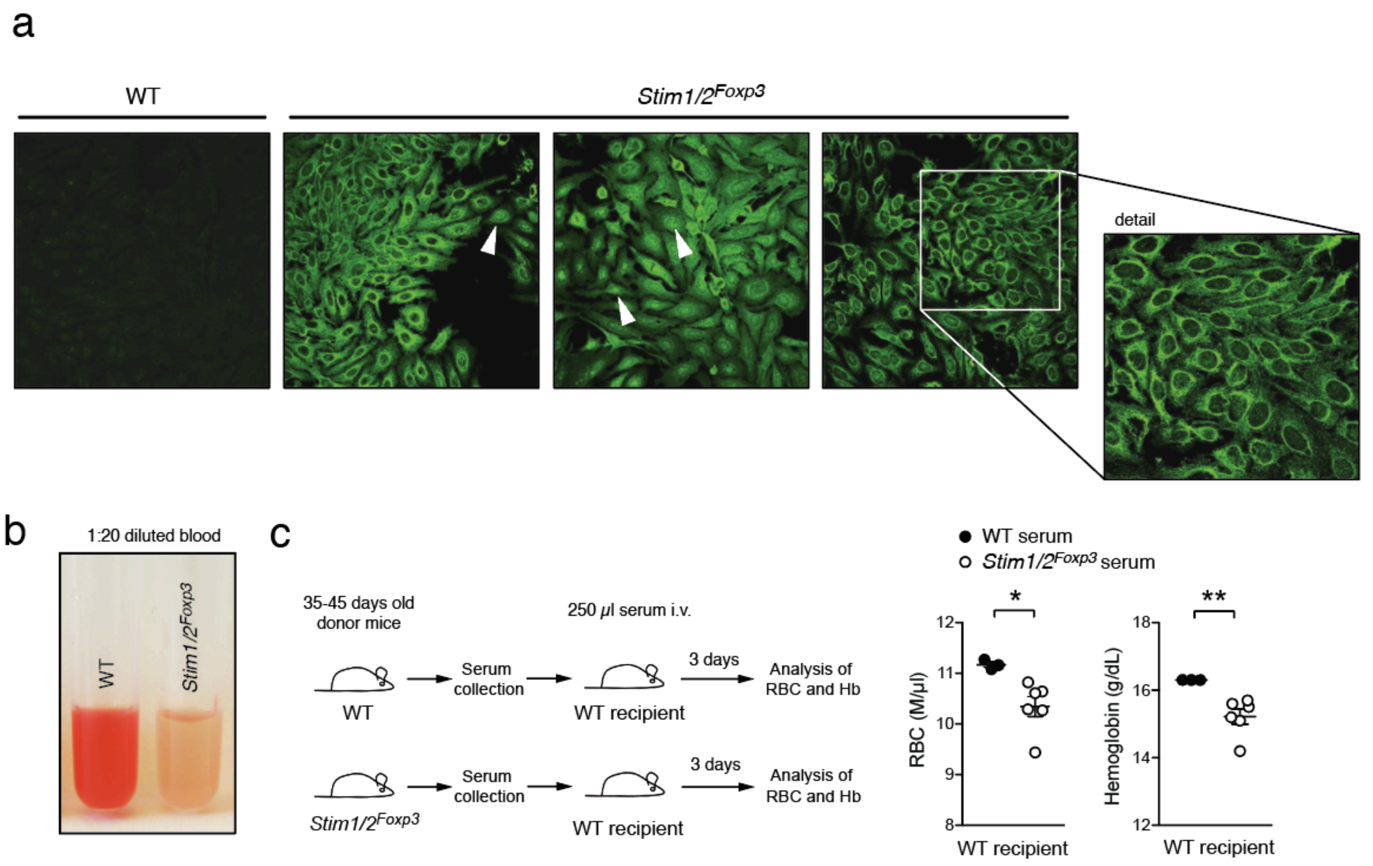


**Supplementary Figure 5. Treg-specific deletion of STIM1 and STIM2 is associated with autoantibody production. (a)** Detection of autoantibodies in the sera of male WT and *Stim1/2$^{Foxp3}$* mice on HEp-2 cells; representative immunofluorescence images of 6 mice. Sera were diluted 1:50. White arrows indicate cells with positive nuclear staining. The nuclear staining pattern indicates the presence of antinuclear antibodies (ANA), whereas the fine fibrous cytosolic staining pattern is consistent with antimitochondrial antibodies (AMA). **(b)** Heparinized blood samples of male WT and *Stim1/2$^{Foxp3}$* mice diluted 1:20 to reveal anemia. **(c)** Transfer of serum from male WT and *Stim1/2$^{Foxp3}$* mice into WT recipient mice. RBC counts and hemoglobin levels in the blood of recipient mice 3 days after serum transfusion; means of 3-6 recipient mice. Each dot represents one mouse. Statistical analysis in c by unpaired Student's t-test. *, $p<0.05$; **, $p<0.01$.

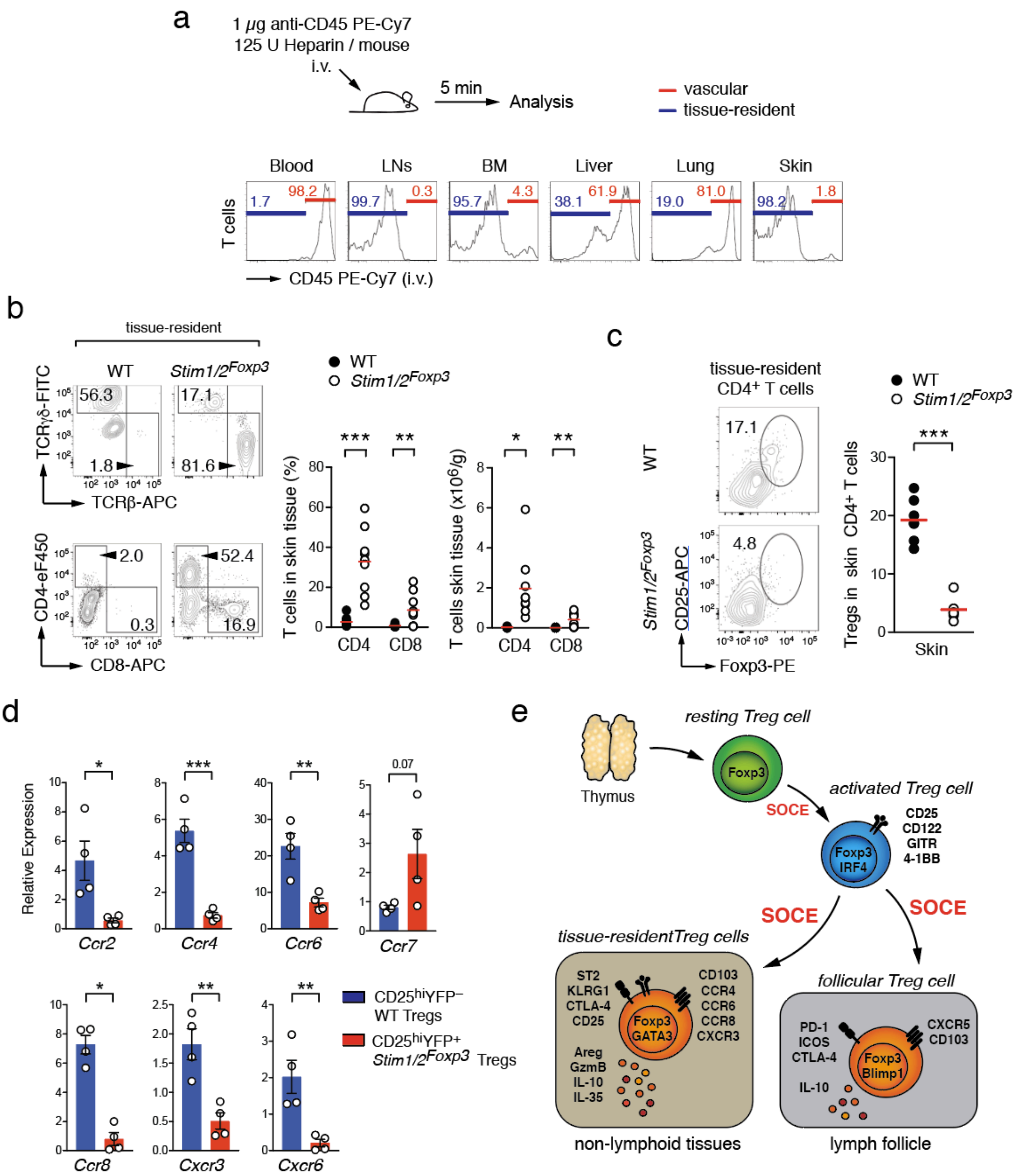


**Supplementary Figure 6. STIM1 and STIM2 regulate tissue-residency of Treg cells and expression of chemokine receptors. (a)** Strategy to identify tissue-resident T cells. Intravenous injection of PE-Cy7 labeled anti-CD45 antibody marks intravascular immune cells whereas parenchymal or tissue-resident cells are not stained. **(b)** Analysis of tissue-resident αβ and γδ T cells (upper panel) and $CD4^+$ and $CD8^+$ T cells (lower panel) in the skin of male WT and *Stim1/2$^{Foxp3}$* mice by flow cytometry; means of 7-9 mice. **(c)** Analysis of tissue-resident $Foxp3^+$ Treg cells in the skin of male WT and *Stim1/2$^{Foxp3}$* mice by flow cytometry; means of 6-7 mice. **(d)** Analysis of *Ccr2, Ccr4, Ccr6, Ccr7, Ccr8, Cxcr3* and *Cxcr6* gene expression in WT and *Stim1/2*-deficient Treg cells isolated from female heterozygous *Stim1/2$^{Foxp3}$* mice by qRT-PCR; means ± SEM of 4 mice. Each dot in b and c represents one mouse. Statistical analysis in b-d by unpaired Student's t-test. *, p<0.05; **, p<0.01, ***, p<0.001. **(e)** Graphical summary how SOCE controls Treg differentiation into Tfr and tissue resident Treg cells. For details see text.

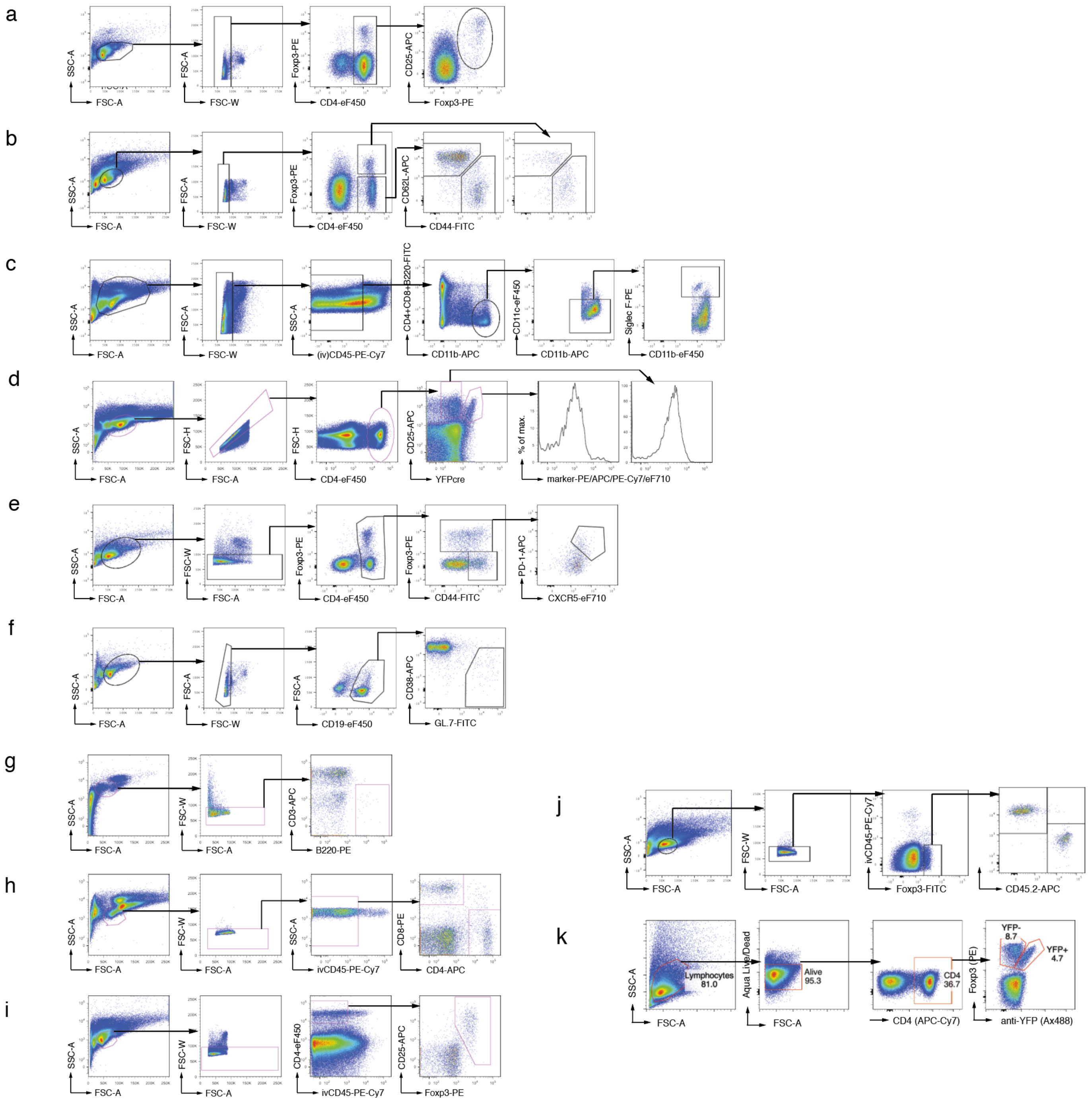


**Supplementary Figure 7. Gating strategies for the flow cytometric detection of immune cells. (a)** Detection of Treg cells in Figure 1c. **(b)** Detection of naïve, effector and memory T cells and Treg cells in Figures 1d and 2b. **(c)** Detection of tissue resident eosinophils in Figure 2f. **(d)** Analysis of expression of transcription factors and cell surface markers on Treg cells in Figures 2c, 3g, 6b and Supplementary Figures 1b,g, 2a and 4d. **(e)** Analysis of CXCR5$^{hi}$ Tfr cells in Figure 4b. **(f)** Determination of the frequency of GC B cells in Figure 4d. **(g)** Strategy to verify B cell depletion after anti-CD20 treatment in Figure 4j. **(h,i)** Identification of tissue resident CD4$^{+}$ and CD8$^{+}$ T cells (h) and Treg cells (i) after *in vivo* labelling of intravascular lymphocytes with anti-CD45-PE-Cy7 in Figures 5c,d, 6a,b and Supplementary Figures 6b,c. **(j)** Analysis of chimerism of tissue resident Treg cells in Figure 6d. **(k)** Determination of the frequency of YFP$^{+}$ and YFP$^{–}$ Treg cells in female heterozygous *Stim1/2*$^{Foxp3}$ mice in Supplementary Figure 1b.

## Supplementary Table 1. Autoimmunity and AIHA in SOCE-deficient patients.

| Mutation | Autoimmunity | Tregs in blood | Outcome | Reference |
|---|---|---|---|---|
| *ORAI1 p.R91W* | n.r. | n.t. | dead (11 mo) | [1, 11, 12, 13] |
| *ORAI1 p.R91W* | ANA, dsDNA autoAbs | reduced | alive after HSCT | [1, 11, 12, 13] |
| *ORAI1 p.A88SfsX25* | Neutropenia, thrombocytopenia | n.t. | dead (11 mo) | [1] |
| *ORAI1 p.A103E/L194P* | n.r. | n.t. | dead after HSCT | [1] |
| *ORAI1 p.H165PfsX1* | n.r. | n.t. | alive after HSCT | [4] |
| *ORAI1 p.R270X* | n.r. | n.t. | dead (7 mo) | [5] |
| *ORAI1 p.V181SfsX8* | n.r. | n.t. | alive after HSCT | [3] |
| *ORAI1 p.L194P* | AIHA, thrombocytopenia | n.t. | dead (8 mo) | [3] |
| *ORAI1 p.G98R* | AIHA, ANA, cardiolipin autoAbs | reduced | alive after HSCT | [3] |
| *ORAI1 p.G98R* | AIHA, thrombocytopenia, antiphospholipid syndrome | n.t. | dead (2.5 yr) | [3] |
| *STIM1 p.E128RfsX9* | AIHA, thrombocytopenia | reduced | dead after HSCT | [6] |
| *STIM1 p.E128RfsX9* | thrombocytopenia | n.t. | alive after HSCT | [6] |
| *STIM1 c.1538-1 G>A* | AIHA | n.t. | dead (2 yr) | [7] |
| *STIM1 p.R429C* | AIHA, thrombocytopenia, ANA | reduced | alive after HSCT | [8] |
| *STIM1 p.R429C* | AIHA, thrombocytopenia | n.t. | dead (21 mo) | [8] |
| *STIM1 p.P165Q* | Lymphadenopathy, splenomegaly | normal | alive (8 yr) | [9] |
| *STIM1 p.P165Q* | n.r. | normal | alive (21 yr) | [9] |
| *STIM1 p.L74P* | thrombocytopenia | normal | alive (11 yr) | [10] |
| *STIM1 p.L74P* | n.r | n.t. | alive (21 yr) | [10] |

## Supplementary Table 2. Antibodies used for flow cytometry.

| Mouse antigen | Clone | Conjugates | Source | Dilution |
|---|---|---|---|---|
| B220 | RA3-6B2 | PE, eFluor450 | eBioscience | 1:400 |
| CD3e | 2C11 | APC, eFluor450 | eBioscience | 1:400 |
| CD4 | GK1.5 | FITC, AlexaFluor488, PE, APC, eFluor450 | eBioscience | 1:400 |
| CD8 | 53-6.7 | FITC, AlexaFluor488, PE, APC, eFluor450 | eBioscience | 1:400 |
| CD11b | M1/70 | eFluor450 | eBioscience | 1:200 |
| CD11c | N418 | APC | Biolegend | 1:200 |
| CD25 | PC61.5 | APC | eBioscience | 1:400 |
| CD38 | 90 | APC | eBioscience | 1:200 |
| CD39 | 24DMS1 | PE-Cy7 | eBioscience | 1:200 |
| CD44 | IM7 | FITC, PE, eFluor450 | eBioscience | 1:400 |
| CD45 | 30-F11 | PE-Cy7 | eBioscience | 1:400 |
| CD45.1 | A20 | FITC, APC, eFluor450 | eBioscience | 1:400 |
| CD45.2 | 104 | PE, APC, eFluor450 | eBioscience | 1:400 |
| CD62L | MEL-14 | APC | eBioscience | 1:400 |
| CD103 | 2 E7 | APC | eBioscience | 1:200 |
| CXCR5 | SPRCL5 | eFluor710 | eBioscience | 1:100 |
| FolR4 | eBio12A5 | APC | eBioscience | 1:200 |
| Foxp3 | FJK-16s | PE, APC | eBioscience | 1:100 |
| GATA3 | TWAJ | APC | eBioscience | 1:100 |
| TCR b | H57-597 | APC | eBioscience | 1:200 |
| TCR gd | UC7-13D5 | FITC | eBioscience | 1:200 |
| GL.7 | GL7 | FITC, AlexaFluor488 | eBioscience | 1:100 |
| ICOS | C398.4A | APC | eBioscience | 1:400 |
| IFNg | XMG1.2 | PE, APC | eBioscience | 1:100 |
| IgG | polyclonal $(Fab)_2$ fragment | FITC | eBioscience | 1:100 |
| IL-17A | eBio17B7 | APC | eBioscience | 1:100 |
| Ki-67 | 16A8 | PE, eFluor450 | eBioscience | 1:200 |
| KLRG1 | 2F1 | eFLuor710 | eBioscience | 1:200 |
| Ox-40 | OX86 | APC | eBioscience | 1:200 |
| PD-1 | RMP1-30 | APC | eBioscience | 1:400 |
| Siglec F | E50-2440 | PE | BD Pharmingen | 1:200 |
| ST2 (IL-33R) | RMST2-2 | APC | eBioscience | 1:50 |
| Ter119 | TER-119 | PE | eBioscience | 1:200 |
| GFP | FM264G | AlexaFluor488 | Biolegend | 1:100 |

**Supplementary Table 3. Primers for realtime PCR.**

| Mouse gene | Forward primer | Reverse primer |
|---|---|---|
| *Foxp3* | CACCCAGGAAAGACAGCAACC | GCAAGAGCTCTTGTCCATTGA |
| *Stim1* | ATTCGGCAAAACTCTGCTTC | GGCCAGAGTCTCAGCCATAG |
| *Stim2* | TCGAAGTGGACGAGAGTGATG | TTTCCACTGTTTCCACAAATCC |
| *Ccr2* | AGCACATGTGGTGAATCCAA | TGCCATCATAAAGGAGCCA |
| *Ccr4* | GGGTACCAGCAGGAGAAGC | CGACGGCATTGCTTCATAG |
| *Ccr6* | TTGAATGGCAGACACTCACAG | GGAGCCTGGATAACCACTGA |
| *Ccr7* | GTCTCTCTCCAGCTAGCCCA | CAAACAGGAGCTGATGTCCA |
| *Ccr8* | GCGGTGAAGAAATCAGGGTA | CTCAGAAGAAAGGCTCGCTC |
| *Cxcr3* | TCTCGTTTTCCCCATAATCG | AGCCAAGCCATGTACCTTGA |
| *Cxcr6* | AAATCTCCCTCGTAGTGCCC | TGGAACAAAGCTACTGGGCT |
| *18S* | CGGCGACGACCCATTCGAAC | GAATCGAACCCTGATTCCCCGT |
| *Gapdh* | TTGATGGCAACAATCTCCAC | CGTCCCGTAGACAAAATGGT |

**Supplementary References**


1. McCarl, C.A. *et al.* ORAI1 deficiency and lack of store-operated Ca2+ entry cause immunodeficiency, myopathy, and ectodermal dysplasia. *J Allergy Clin Immunol* **124**, 1311-1318 e1317 (2009).
2. Vaeth, M. *et al.* Store-Operated Ca Entry in Follicular T Cells Controls Humoral Immune Responses and Autoimmunity. *Immunity* (2016).
3. Lian, J. *et al.* ORAI1 mutations abolishing store-operated Ca(2+) entry cause anhidrotic ectodermal dysplasia with immunodeficiency. *J Allergy Clin Immunol* (2017).
4. Chou, J. *et al.* A novel mutation in ORAI1 presenting with combined immunodeficiency and residual T-cell function. *J Allergy Clin Immunol* **136**, 479-482 e471 (2015).
5. Badran, Y.R. *et al.* Combined immunodeficiency due to a homozygous mutation in ORAI1 that deletes the C-terminus that interacts with STIM 1. *Clin Immunol* **166-167**, 100-102 (2016).
6. Picard, C. *et al.* STIM1 mutation associated with a syndrome of immunodeficiency and autoimmunity. *N Engl J Med* **360**, 1971-1980 (2009).
7. Byun, M. *et al.* Whole-exome sequencing-based discovery of STIM1 deficiency in a child with fatal classic Kaposi sarcoma. *J Exp Med* **207**, 2307-2312 (2010).
8. Fuchs, S. *et al.* Antiviral and regulatory T cell immunity in a patient with stromal interaction molecule 1 deficiency. *J Immunol* **188**, 1523-1533 (2012).
9. Schaballie, H. *et al.* A novel hypomorphic mutation in STIM1 results in a late-onset immunodeficiency. *J Allergy Clin Immunol* **136**, 816-819 e814 (2015).
10. Parry, D.A. *et al.* A homozygous STIM1 mutation impairs store-operated calcium entry and natural killer cell effector function without clinical immunodeficiency. *J Allergy Clin Immunol* **137**, 955-957 e958 (2016).
11. Feske, S. et al. A mutation in Orai1 causes immune deficiency by abrogating CRAC channel function. *Nature* **441**, 179-85 (2006).
12. Feske, S. et al. The duration of nuclear residence of NFAT determines the pattern of cytokine expression in human SCID T cells. *J Immunol*. **165**, 297-305 (2000).
13. Feske, S. et al. Severe combined immunodeficiency due to defective binding of the nuclear factor of activated T cells in T lymphocytes of two male siblings. *Eur J Immunol*. **26**, 2119-26 (1996).
