# Supplementary material for: Tissue resident and follicular Treg cell differentiation is regulated by CRAC channels
Source: Nat Commun. 2019 Mar 12;10:1183. doi: 10.1038/s41467-019-08959-8 (PMC6414608; doi:10.1038/s41467-019-08959-8)
Supplement: Supplementary file 3 — Reporting Summary [file 41467_2019_8959_MOESM3_ESM.pdf]

nature research

Corresponding author(s): Dr. Stefan Feske
manuscript NCOMMS-18-30624-T

Reporting Summary

Nature Research wishes to improve the reproducibility of the work that we publish. This form provides structure for consistency and transparency in reporting. For further information on Nature Research policies, see Authors & Referees and the Editorial Policy Checklist.

Statistical parameters

When statistical analyses are reported, confirm that the following items are present in the relevant location (e.g. figure legend, table legend, main text, or Methods section).

n/a	Confirmed	
☐	☒	The exact sample size (*n*) for each experimental group/condition, given as a discrete number and unit of measurement
☐	☒	An indication of whether measurements were taken from distinct samples or whether the same sample was measured repeatedly
☐	☒	The statistical test(s) used AND whether they are one- or two-sided<br>*Only common tests should be described solely by name; describe more complex techniques in the Methods section.*
☒	☐	A description of all covariates tested
☐	☒	A description of any assumptions or corrections, such as tests of normality and adjustment for multiple comparisons
☐	☒	A full description of the statistics including central tendency (e.g. means) or other basic estimates (e.g. regression coefficient) AND variation (e.g. standard deviation) or associated estimates of uncertainty (e.g. confidence intervals)
☐	☒	For null hypothesis testing, the test statistic (e.g. *F*, *t*, *r*) with confidence intervals, effect sizes, degrees of freedom and *P* value noted<br>*Give P values as exact values whenever suitable.*
☒	☐	For Bayesian analysis, information on the choice of priors and Markov chain Monte Carlo settings
☒	☐	For hierarchical and complex designs, identification of the appropriate level for tests and full reporting of outcomes
☒	☐	Estimates of effect sizes (e.g. Cohen's *d*, Pearson's *r*), indicating how they were calculated
☐	☒	Clearly defined error bars<br>*State explicitly what error bars represent (e.g. SD, SE, CI)*

Our web collection on statistics for biologists may be useful.

Software and code

Policy information about availability of computer code

Data collection	Flow cytometry data was acquired using FACSDiva (BD Pharmingen) and analyzed using the FlowJo software (TreeStar).<br>IF microscopy data was acquired with NIS Elements BR 3.2 (Nikon) software.<br><br>RNA quality and quantity was analyzed on a Bioanalyzer 2100 (Agilent) using a PICO chip. RNA-seq libraries were pooled equimolarly, and loaded on the HiSeq 2500 Sequencing System (Illumina) and run as single 50 nucleotide reads<br><br>All data was visualized with Microsoft excel or Graphpad Prism and statistical analysis was done using the Prism Software package (Graphpad).
Data analysis	For gene expression analysis, reads were aligned to the NCBIM37 (iGenome) mouse genome using Bowtie software (Version 1.0.0) (83) with 2 mismatches allowed. Uniquely-mapped reads were further processed by removing PCR duplicates with Picard MarkDuplicates (http://broadinstitute.github.io/picard/). Transcripts were counted using HTSeq and differential gene expression between WT and Stim1/2-deficient Treg cells was performed separately for each cell type using DESeq2 (84) Bioconductor package in the R statistical programming environment. Differences in gene expression were considered significant if padj <0.05. Gene expression signatures and canonical pathway analyses and were performed using Ingenuity's Pathway Analysis (IPA, Qiagen), database for annotation, visualization and integrated discovery (DAVID, https://david.ncifcrf.gov) and gene set enrichment analysis (GSEA, https://software.broadinstitute.org/gsea/index.jsp). For network cluster analyses, positively and negatively regulated gene identifiers were used to generate enrichment maps in Cytoscape (http://www.cytoscape.org). Heatmaps of selected genes were created using the conditional formatting tool in Microsoft Excel.

All data was visualized with Microsoft Excel and Graphpad Prism and statistical analysis was done using the Prism Software (Graphpad).

For manuscripts utilizing custom algorithms or software that are central to the research but not yet described in published literature, software must be made available to editors/reviewers upon request. We strongly encourage code deposition in a community repository (e.g. GitHub). See the Nature Research guidelines for submitting code & software for further information.

Data

Policy information about availability of data

All manuscripts must include a data availability statement. This statement should provide the following information, where applicable:

- Accession codes, unique identifiers, or web links for publicly available datasets
- A list of figures that have associated raw data
- A description of any restrictions on data availability

GEO accession code for RNA-seq data:

Field-specific reporting

Please select the best fit for your research. If you are not sure, read the appropriate sections before making your selection.

☒ Life sciences ☐ Behavioural & social sciences ☐ Ecological, evolutionary & environmental sciences

For a reference copy of the document with all sections, see nature.com/authors/policies/ReportingSummary-flat.pdf

Life sciences study design

All studies must disclose on these points even when the disclosure is negative.

Sample size	Sample sizes were based on experience and experimental complexity. Furthermore, fundamental findings were confirmed with a variety of different methods to avoid any method-specific bias.<br>In addition, each figure legend contains the information how many samples/mice were used for the described experiment.
Data exclusions	No data were excluded.
Replication	All experiments were done at least in two independent experiments. Numbers of mice or samples per experiment was generally > 2
Randomization	No randomization was used.
Blinding	The Investigators were not blinded to the experimental design.

Reporting for specific materials, systems and methods

Materials & experimental systems

n/a	Involved in the study
☐	☐ Unique biological materials
☐	☒ Antibodies
☐	☐ Eukaryotic cell lines
☐	☐ Palaeontology
☐	☒ Animals and other organisms
☐	☐ Human research participants

Methods

n/a	Involved in the study
☐	☐ ChIP-seq
☐	☒ Flow cytometry
☐	☐ MRI-based neuroimaging

Unique biological materials

Policy information about availability of materials

Obtaining unique materials	Stim1fl/fl Stim2 fl/fl Foxp3-YFP-cre mice (see materials and methods for details). No other unique biological material was used.

Antibodies

Antibodies used | A full list of antibodies and primers is also provided in the supplemental information. The following antibodies for flow cytometry were used in this study:
Mouse antigen, Clone, Conjugates, Source
B220, RA3-6B2, PE, eFluor450, eBiosciences
CD3e, 2C11, APC, eFluor450, eBiosciences
CD4, GK1.5 FITC, AlexaFluor488, PE, APC, eFluor450, eBiosciences
CD8, 53-6.7 FITC, AlexaFluor488, PE, APC, eFluor450, eBiosciences
CD11b, M1/70, eFluor450, eBiosciences
CD11c, N418, APC, Biolegend
CD25, PC61.5, APC, eBiosciences
CD38, 90, APC, eBiosciences
CD39, 24DMS1, PE-Cy7, eBiosciences
CD44, IM7, FITC, PE, eFluor450, eBiosciences
CD45, 30-F11, PE-Cy7, eBiosciences
CD45.1, A20 , FITC, APC, eFluor450, eBiosciences
CD45.2, 104, PE, APC, eFluor450, eBiosciences
CD62L, MEL-14, APC, eBiosciences
CD103, 2 E7, APC, eBiosciences
CXCR5, SPRCL5, eFluor710, eBiosciences
FolR4, eBio12A5, APC, eBiosciences
Foxp3, FJK-16s PE, APC, eBiosciences
GATA3, TWAJ, APC, eBiosciences
TCRb, H57-597, APC, eBiosciences
TCRgd, UC7-13D5, FITC, eBiosciences
GL.7, GL7, FITC, AlexaFluor488, eBiosciences
ICOS, C398.4A, APC, eBiosciences
IFNg, XMG1.2, PE, APC, eBiosciences
IgG, polyclonal (Fab)2 fragment, FITC, eBiosciences
IL-17A, eBio17B7, APC, eBiosciences
Ki-67, 16A8 PE, eFluor450, eBiosciences
KLRG1, 2F1, eFLuor710, eBiosciences
Ox-40, OX86, APC, eBiosciences
PD-1, RMP1-30, APC, eBiosciences
Siglec F, E50-2440, PE, BD Pharmingen
ST2 (IL-33R), RMST2-2, APC, eBiosciences
Ter119, TER-119, PE, eBiosciences

Validation | All antibodies listed above were purchased commercially and tested in the lab for their performance.

Eukaryotic cell lines

Policy information about cell lines

Cell line source(s) | no cell lines were used.

Authentication | n/a

Mycoplasma contamination | n/a

Commonly misidentified lines (See ICLAC register) | n/a

Palaeontology

Specimen provenance | n/a

Specimen deposition | n/a

Dating methods | n/a

☐ Tick this box to confirm that the raw and calibrated dates are available in the paper or in Supplementary Information.

Animals and other organisms

Policy information about studies involving animals; ARRIVE guidelines recommended for reporting animal research

Laboratory animals | Stim1fl/fl and Stim2fl/fl mice have been described before (Oh hora et al. Nat immunol; 2008). Foxp3-YFPcre (B6.129(Cg)-

Laboratory animals	Foxp3tm4(YFP/icre)Ayr/J; JAX strain 016959), Rag1–/– (B6.129S7-Rag1tm1Mom/J; JAX strain 002216) and congenic CD45.1+ mice (B6.SJL-Ptprca Pepcb/BoyJ; JAX strain 002014) were purchased from The Jackson Laboratory. Crossing Stim1fl/flStim2fl/fl mice to Foxp3-YFPcre animals generated mice with Treg-specific deletion of Stim1/2 genes (Stim1fl/flStim2fl/fl Foxp3-YFPcre or Stim1/2Foxp3 mice for short). All animals were maintained on a pure C57BL/6 genetic background and housed under SPF conditions in accordance with the institutional guidelines for animal welfare approved by the IACUC at NYU School of Medicine. Male and female mice of different age were used in this study as indicated in the figure legends.
Wild animals	no wild animals were used.
Field-collected samples	no field collected samples were used.

Human research participants

Policy information about studies involving human research participants

Population characteristics	n/a
Recruitment	n/a

ChIP-seq

Data deposition

☐ Confirm that both raw and final processed data have been deposited in a public database such as GEO.

☐ Confirm that you have deposited or provided access to graph files (e.g. BED files) for the called peaks.

Data access links<br>*May remain private before publication.*	n/a
Files in database submission	n/a
Genome browser session<br>(e.g. UCSC)	n/a

Methodology

Replicates	n/a
Sequencing depth	n/a
Antibodies	n/a
Peak calling parameters	n/a
Data quality	n/a
Software	n/a

Flow Cytometry

Plots

Confirm that:

☒ The axis labels state the marker and fluorochrome used (e.g. CD4-FITC).

☒ The axis scales are clearly visible. Include numbers along axes only for bottom left plot of group (a 'group' is an analysis of identical markers).

☒ All plots are contour plots with outliers or pseudocolor plots.

☒ A numerical value for number of cells or percentage (with statistics) is provided.

Methodology

Sample preparation	Staining of cell surface or intracellular antigens with fluorescently labeled antibodies was carried out on ice. Samples were acquired on a LSR II flow cytometer (BD Biosciences) and analyzed using the FlowJo software (TreeStar). Purification of various immune cell populations was performed on a SY3200 cell sorter (Sony) using a 70 µm nozzle.<br>To label intravascular immune cells in situ, mice were injected retroorbitally with 1 µg anti-CD45 PE-Cy7 (clone 30-F11) together with 125 U Heparin in 100 µl PBS. After 5 min, mice were sacrificed and lymphoid and non-lymphoid organs were extracted. Skin, lung and liver tissue (ca. 500 mg) was minced in 2 mm pieces and digested in for 20 min at 37 ˚C using 500 µg/ml Liberase TL (lung, skin) or 25 µg/ml Liberase TM (liver, both Roche) in 5 ml FBS-free RPMI medium. Following digestion, lysates were washed twice and the lymphocyte fraction was enriched by 35% percoll gradient centrifugation before analysis by flow cytometry.

Instrument	LSR II flow cytometer (BD Biosciences) and SY3200 cell sorter (Sony)
Software	Acquisition was done using the FACSDiva software (BD Biosciences) and final analysis was performed using the FlowJo software (TreeStar).
Cell population abundance	Cell populations were analyzed by gating on live lymphocytes and single cell fractions. Depending on the experimental question relative or absolute numbers were calculated. A representative example of flow cytometry data (dot plots) and the summary of several independent experiments (e.g. bar graphs) is provided in the figures.
Gating strategy	Cell populations were analyzed by gating on live lymphocytes (FSC/SSC) and single cell fractions (FSC-A/FSC-H/FSC-W). Depending on the experimental question, different gating strategies were used and displayed in the supplemental information and/or indicated in the respective figure (e.g. CD4+ cells gated; Fig. 1c)

☒ Tick this box to confirm that a figure exemplifying the gating strategy is provided in the Supplementary Information.

Magnetic resonance imaging

Experimental design

Design type	n/a
Design specifications	n/a
Behavioral performance measures	n/a

Acquisition

Imaging type(s)	n/a
Field strength	n/a
Sequence & imaging parameters	n/a
Area of acquisition	n/a

Diffusion MRI ☐ Used ☐ Not used

Preprocessing

Preprocessing software	n/a
Normalization	n/a
Normalization template	n/a
Noise and artifact removal	n/a
Volume censoring	n/a

Statistical modeling & inference

Model type and settings	n/a
Effect(s) tested	n/a

Specify type of analysis: ☐ Whole brain ☐ ROI-based ☐ Both

Statistic type for inference (See Eklund et al. 2016)	n/a
Correction	n/a

Models & analysis

n/a	Involved in the study
☒	☐ Functional and/or effective connectivity
☒	☐ Graph analysis
☒	☐ Multivariate modeling or predictive analysis
